# *FLEXR* GUI: a graphical user interface for multi-conformer modeling of proteins

**DOI:** 10.1107/S1600576724001523

**Published:** 2024-03-27

**Authors:** Timothy R. Stachowski, Marcus Fischer

**Affiliations:** a Department of Chemical Biology and Therapeutics, St Jude Children’s Research Hospital, Memphis, TN 38105, USA; Oak Ridge National Laboratory, USA; North Carolina State University, USA

**Keywords:** model building, multi-conformer models, flexibility, conformational heterogeneity, graphical user interfaces, *FLEXR*, *Coot*, proteins, electron-density maps

## Abstract

Proteins are well known ‘shapeshifters’ which change conformation to function. In crystallography, multiple conformational states are often present within the crystal and the resulting electron-density map. Yet, explicitly incorporating alternative states into models to disentangle multi-conformer ensembles is challenging. We previously reported the tool *FLEXR*, which, within a few minutes, automatically separates conformational signal from noise and builds the corresponding, often missing, structural features into a multi-conformer model. To make the method widely accessible for routine multi-conformer building as part of the computational toolkit for macromolecular crystallography, we present a graphical user interface (GUI) for *FLEXR*, designed as a plugin for *Coot 1*. The GUI implementation seamlessly connects *FLEXR* models with the existing suite of validation and modeling tools available in *Coot*. We envision that *FLEXR* will aid crystallographers by increasing access to a multi-conformer modeling method that will ultimately lead to a better representation of protein conformational heterogeneity in the Protein Data Bank. In turn, deeper insights into the protein conformational landscape may inform biology or provide new opportunities for ligand design. The code is open source and freely available on GitHub at https://github.com/TheFischerLab/FLEXR-GUI.

## Abstract 2

This article describes a graphical user interface (GUI) for the multi-conformer modeling program *FLEXR*, designed as a plugin within *Coot 1*. At the click of a button, the GUI allows seamless integration of *FLEXR* into the crystallographic modeling and validation pipeline within *Coot*.

## 1. Introduction

Proteins are dynamic machines that change conformation to perform functions such as catalysis (Eisenmesser *et al.*, 2005 ▸), allostery (Buhrman *et al.*, 2010 ▸) and ligand binding (Krojer *et al.*, 2020 ▸). These changes occur across timescales and can be global and slow, such as major domain or hinge motions, or local and fast, such as subtle changes in the rotameric state of individual amino acid residues (Henzler-Wildman & Kern, 2007 ▸). The protein conformational landscape can also be altered (St-Jacques *et al.*, 2023 ▸) by changes in the environment, such as temperature (Stachowski *et al.*, 2022 ▸; Wolff *et al.*, 2023 ▸). Detailing the course of these conformational changes along the protein functional life cycle, for example through time-resolved crystallography (Pearson & Mehrabi, 2020 ▸; Kupitz *et al.*, 2014 ▸), can reveal deeper biological insights (Kang *et al.*, 2015 ▸; Buhrman *et al.*, 2010 ▸; Johnson *et al.*, 2019 ▸) and new features to leverage for drug design (Carlson, 2002 ▸; Kim *et al.*, 2023 ▸).

X-ray crystallography is still the foremost method to determine three-dimensional protein crystal structures with high spatial resolution, often better than 2 Å. While structures solved using crystallography are often simplified to representations as single models, this ignores their dynamic nature and subtle yet important alternative states (Bourne *et al.*, 2022 ▸). Rather, explicitly modeling multiple protein conformations that are present throughout the crystal and the resulting electron-density map promises richer insights into the protein conformational landscape. Yet, even at high resolutions (better than 2 Å), detecting these conformations is challenging since minor states are often masked by more predominate ones. Additionally, genuine signal may be obscured by noise (Lang *et al.*, 2010 ▸) or mistaken as ordered water molecules, which can concurrently alternate with corresponding changes in the protein (Lang *et al.*, 2014 ▸). Few tools exist that can disentangle whether these electron-density features represent signal or noise (Lang *et al.*, 2010 ▸) or that are able to discern the specific combination of states that best explain the density (Riley *et al.*, 2021 ▸; Burnley *et al.*, 2012 ▸; Wankowicz *et al.*, 2023 ▸).

We previously reported *FLEXR*, a tool that creates multi-state models by sampling electron density around side-chain dihedral angles and maps that signal to specific rotameric states (Stachowski & Fischer, 2023 ▸). Alternate states are then explicitly added to models using *Coot* building tools (Casañal *et al.*, 2020 ▸). We showed that *FLEXR* finds previously unappreciated conformational states in ligand binding sites and produces multi-state models without compromising model quality (Stachowski & Fischer, 2023 ▸). *FLEXR* was first released as a command-line interface (CLI) tool. Here, we present a graphical user interface (GUI) with a point-and-click menu to run the entire *FLEXR* workflow. To show the ease and accessibility of the *FLEXR* GUI, we interrogated a typical use case, a ‘straight from the Protein Data Bank’ (PDB) structure of the flexible therapeutic target KRAS. In less than a minute, *FLEXR* generated a multi-state model that not only reproduced all flexible sites in the deposited model but also revealed previously missed side-chain conformations. Having the GUI available as a *Coot* plugin allows the user to immediately and interactively interrogate *FLEXR* models with the extensive validation and modeling toolboxes available within *Coot*.

## 2. *FLEXR* GUI

### 2.1. Implementation, availability and installation

*FLEXR* is implemented in Python 3.9 and the GUI was designed using the toolkit *GTK*4 (GTK Team, 2023 ▸), the same one as used by *Coot 1* (Emsley, 2023 ▸). Python was chosen because of its simplicity and the potential to integrate with other structural-biology programs, many of which are written in Python. *Phenix* (Liebschner *et al.*, 2019 ▸) is required since it is used to run *Ringer* (Lang *et al.*, 2010 ▸), which is available in the *mmtbx* library (https://cctbx.github.io/mmtbx/mmtbx.html). To ensure consistent column labels and follow the original *Ringer* protocol (Fraser *et al.*, 2011 ▸), map coefficients are recalculated from deposited reflections with *phenix.maps* prior to running *Ringer*. *FLEXR* starts from a single-conformer model; if alternative side-chain conformations are present these are removed using phenix.pdbtools remove_alt_confs = True. Otherwise, all *Phenix* steps are run with default settings. At the click of a single button, the *FLEXR* GUI interfaces with these programs to perform the entire process from calculating map coefficients and computing electron densities to model building. Complete instructions for downloading, installing and using the GUI version of *FLEXR* are documented in the supporting information and at https://github.com/TheFischerLab/FLEXR-GUI. For the tutorial, *Coot 1* (Emsley, 2023 ▸) should be installed using *Homebrew* (https://brew.sh/). *FLEXR* is not backwards compatible with earlier versions of *Coot*. The *FLEXR* GUI was tested on Macs with either Intel or silicon processor architectures running macOS Sonoma. Future development will focus on making *FLEXR* available on Linux and Windows operating systems.

### 2.2. Functional description

#### 2.2.1. Evaluating electron density

**Figure 1:**
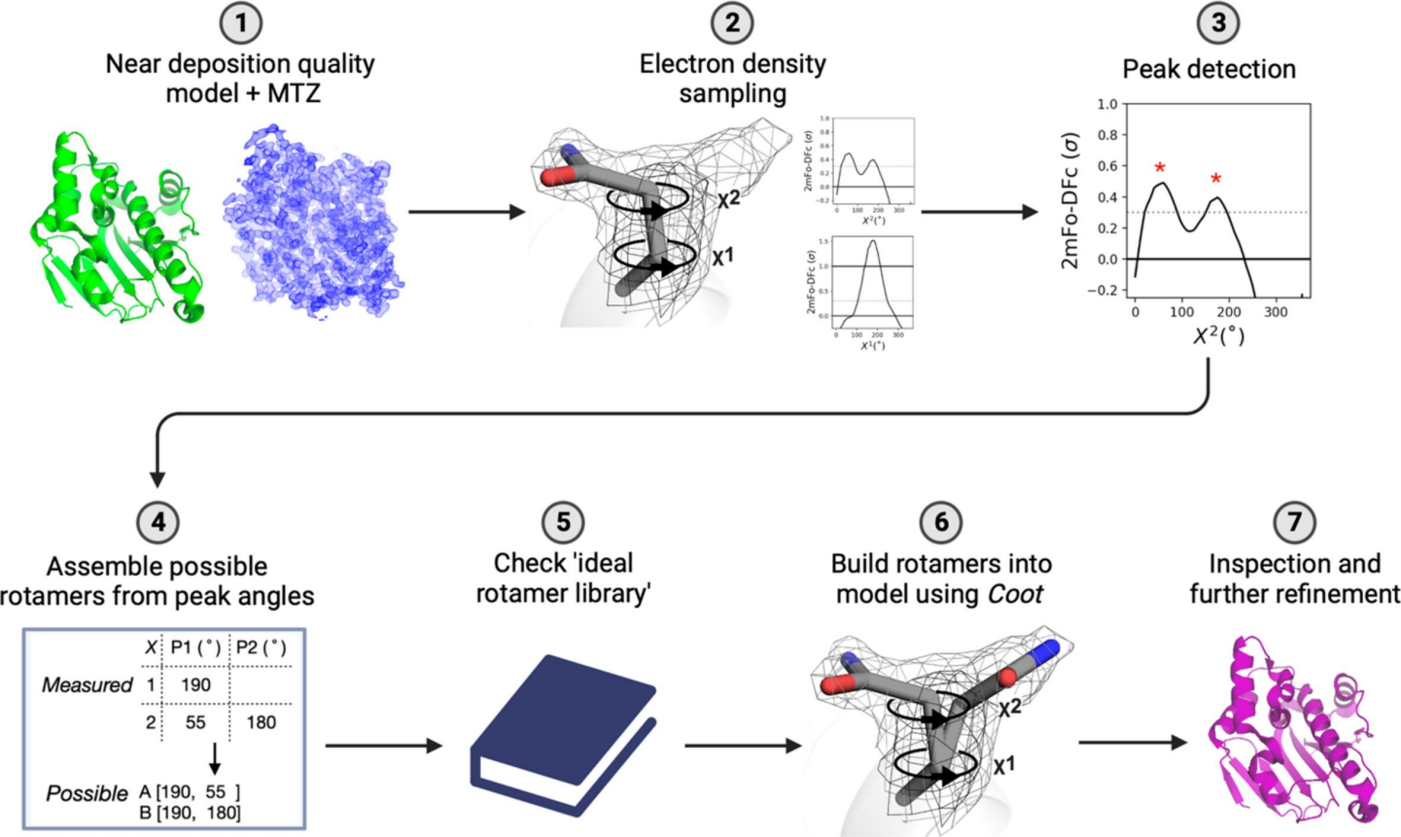
*FLEXR* workflow. (1) Users should start with a near deposition quality model that has solvent modeled in PDB or mmCIF format and an MTZ file containing reflections and map coefficients. (2) *Ringer* is used to compute electron density around side-chain dihedral angles. (3) Electron densities above a user-defined threshold (usually 0.3σ; dotted line) are evaluated for peaks (red stars). Peak intensities and rotation angles are recorded. (4) Peaks detected around the dihedral angles of each side chain are assembled across dihedral angles into possible rotamers. (5) These combinations are checked against the ideal rotamer library (Lovell *et al.*, 2000 ▸) to reject outliers and to determine which rotamer the series of angles corresponds to. (6) Rotamers that pass this check are built into models using *Coot*. (7) The final *FLEXR* models are ready for manual inspection and refinement before deposition.

The inputs to the *FLEXR* GUI are coordinates (PDB or mmCIF) and an MTZ file containing reflections and map coefficients. The model should be near deposition quality [Fig. 1 ▸(1)]. It is crucial that *FLEXR* has a solid starting point to explore all dihedral angles at maximum signal-to-noise ratio in the electron-density map. *Ringer* is used to sample electron density around each dihedral angle of each side chain in the input model (Lang *et al.*, 2010 ▸) [Fig. 1 ▸(2)]. *FLEXR* uses the *find_peaks* function from the *SciPy* library (Virtanen *et al.*, 2020 ▸) to find heightened levels of electron density above a user-defined threshold (default 0.3σ) [Fig. 1 ▸(3)]. The angle at which each electron-density peak occurs is recorded for each dihedral angle of a residue. If the ‘*Ringer* plotting’ option is selected, these plots can be used to monitor peak-detection accuracy [Fig. 1 ▸(3)].

#### 2.2.2. Determining rotamers

For each side chain, all possible combinations of angles for each peak detected in Section 2.2.1 are assembled across all dihedral angles [Fig. 1 ▸(4)]. Each combination is then cross-checked against the ‘ideal rotamer library’ (Lovell *et al.*, 2000 ▸) [Fig. 1 ▸(5)]. Combinations that have tolerable geometry (within 30°) are assigned a ‘rotamer name’ from the matching entry; for instance, ‘p’, ‘t’ or ‘m’ for the three ideal threonine rotamers. Where a measured rotamer matches multiple entries in the rotamer library, the rotamer that occurs most often, as surveyed in the ideal rotamer library, is selected for building.

#### 2.2.3. Multi-conformer model building

Rotamers are automatically built into models using *Coot* building functions [Fig. 1 ▸(6)] and displayed directly in the *Coot* workspace. The output models should be further inspected and refined prior to deposition [Fig. 1 ▸(7)]. An update from the first release of *FLEXR* is that rotamers for a particular residue are added to the model in decreasing order of electron-density signal strength so that rotamers with stronger signal are added to the model first. The signal strength for a potential rotamer is the sum of the relative peak intensities across all dihedral angles. This is important as the new option ‘Max. number of alts/residue’ allows users to define the maximum number of conformers built per residue for an entire structure (default = 3). By setting this constraint to a lower value, fewer conformers will potentially be added overall and the model will have fewer low-signal conformers. Likewise, setting this constraint to a higher value will allow more conformers to be added to the model, which will have lower signal and typically be refined to lower occupancy. After inspecting the *FLEXR* model, the GUI makes it easy for users to test different settings and produce an internally consistent model that maximally captures weak density features of interest.

### 2.3. Graphical user interface

**Figure 2:**
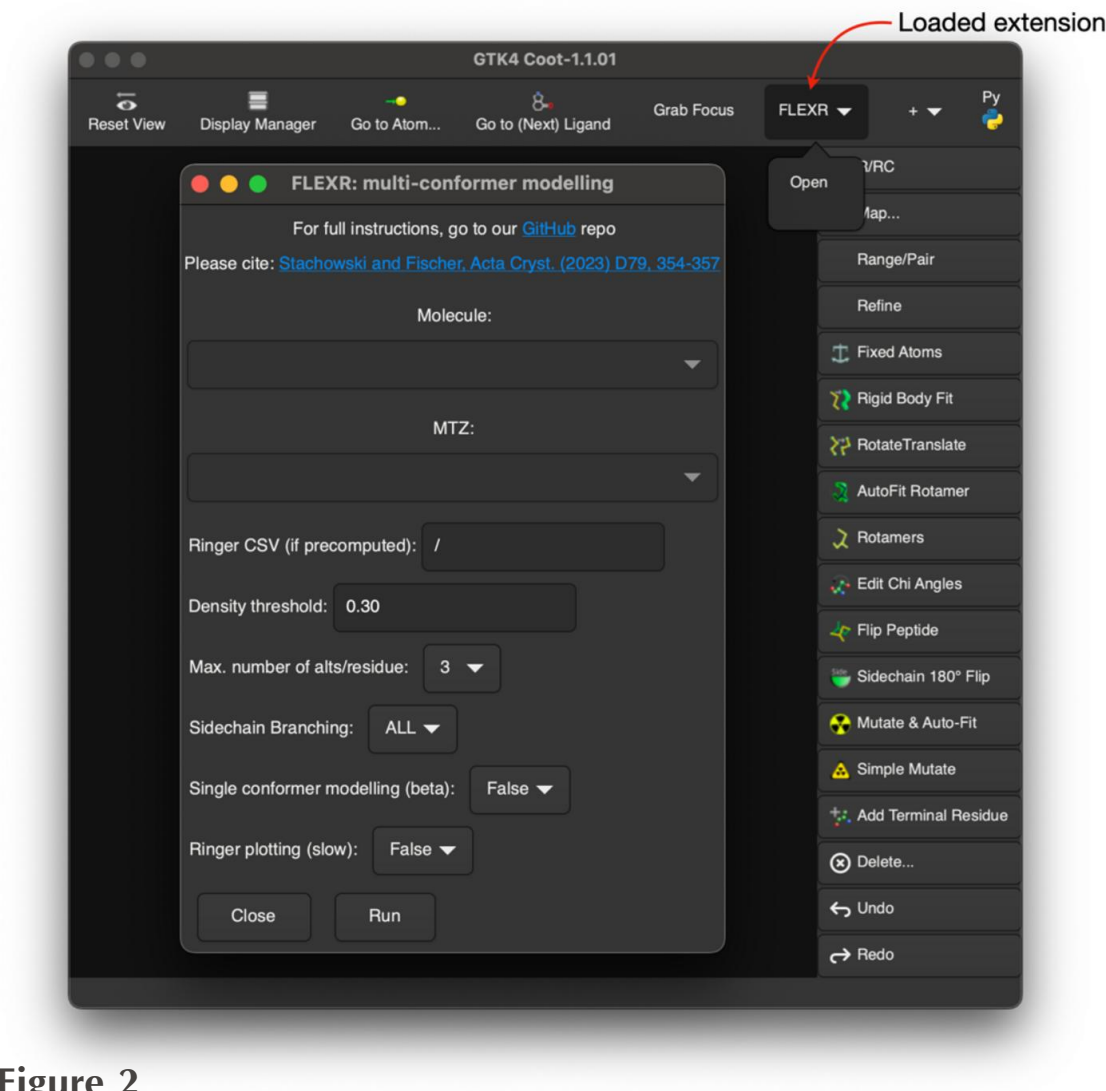
The *FLEXR* GUI. Once loaded, the *FLEXR* extension is available from the top menu bar in *Coot 1* (red arrow).

*FLEXR* was initially released as a tool available only through the CLI (Stachowski & Fischer, 2023 ▸). To expand *FLEXR*’s use, the GUI version presented here gives users a point-and-click method to access some of the same options and outputs available in the CLI version (Fig. 2 ▸; see also Section S1.2 of the supporting information, point 6). This overcomes potential bottlenecks of the CLI version, which requires several independent steps including providing pre-computed *Ringer* densities and providing a single-conformer model as input. The GUI option seamlessly performs these steps, given that the user has *Phenix* installed. The GUI is loaded as a *Coot 1* extension where it is available in the top menu bar. The GUI was designed to match the look and feel of *Coot* so that it is intuitive to crystallographers familiar with *Coot*. Users can start *FLEXR* in the GUI by simply providing a coordinate file (PDB or mmCIF) and an MTZ file containing map coefficients and reflections. As part of the workflow, map coefficients will first be recalculated with *Phenix* to ensure consistent column labeling and then *Ringer* will compute electron densities. Alternatively, users have the option to supply pre-computed *Ringer* densities, for instance if they want to use a different map such as an OMIT map to minimize model bias (Terwilliger *et al.*, 2008 ▸). The interface also gives users options to control some of the *FLEXR* decision making via the following parameters: the electron-density threshold for peak finding (default = 0.3σ), the maximum number of conformers to build for each residue (default = 3), whether to add atoms with alternative conformations for the entire residue or just the side chain starting at the Cα atom (default = ALL), and the option to produce plots of electron-density values with peak-finding information (default = False). *FLEXR* produces a multi-state model and a single-state model, both of which are shown in *Coot* alongside the original model. By using the many validation and modeling tools native to *Coot*, users can then inspect and further alter *FLEXR* models or repeat the process with different settings. A tutorial on using the *FLEXR* GUI is provided in the supporting information and on GitHub.

## 3. Results

To illustrate the utility of the GUI, we applied *FLEXR* to a recently reported structure of KRAS-G12D bound to an inhibitor (PDB ID 8txg, resolution 1.5 Å; Cheng *et al.*, 2023 ▸). In addition to its therapeutic significance (Punekar *et al.*, 2022 ▸), KRAS is known to be structurally dynamic (Ostrem *et al.*, 2013 ▸) and has four residues modeled with alternative conformations at a resolution of 1.5 Å. KRAS features a P-loop containing residues G12 and G13, which are typically mutated in cancers, and has two highly dynamic Switch I and II regions that control substrate and inhibitor binding, respectively (Ostrem *et al.*, 2013 ▸). Using the GUI, we tested whether *FLEXR* could reproduce alternative conformations in the deposited model and find additional sites of flexibility. We downloaded coordinates and the 2*F*_o_ − *F*_c_ map coefficients and reflections directly from the PDBe (https://www.ebi.ac.uk/pdbe/) using the ‘Fetch PDB & Map using EDS…’ function in *Coot*. We ran *FLEXR* from the GUI using default settings, which took ∼24 s to recalculate map coefficients, 3 s to calculate *Ringer* electron-density values and 13 s to build the *FLEXR* multi-conformer model. The entire process to produce a *FLEXR* model took ∼40 s. If desired, the user can also create plots of *Ringer* values, which in this case took an additional 50 s. Refinement often improves the fit of the terminal atoms of ideal rotamers in the electron density and yields occupancy information for the relative populations of alternative states. To this end, both deposited and *FLEXR* models were refined with three macrocycles of *Phenix* using default settings and without solvent updating.

**Figure 3:**
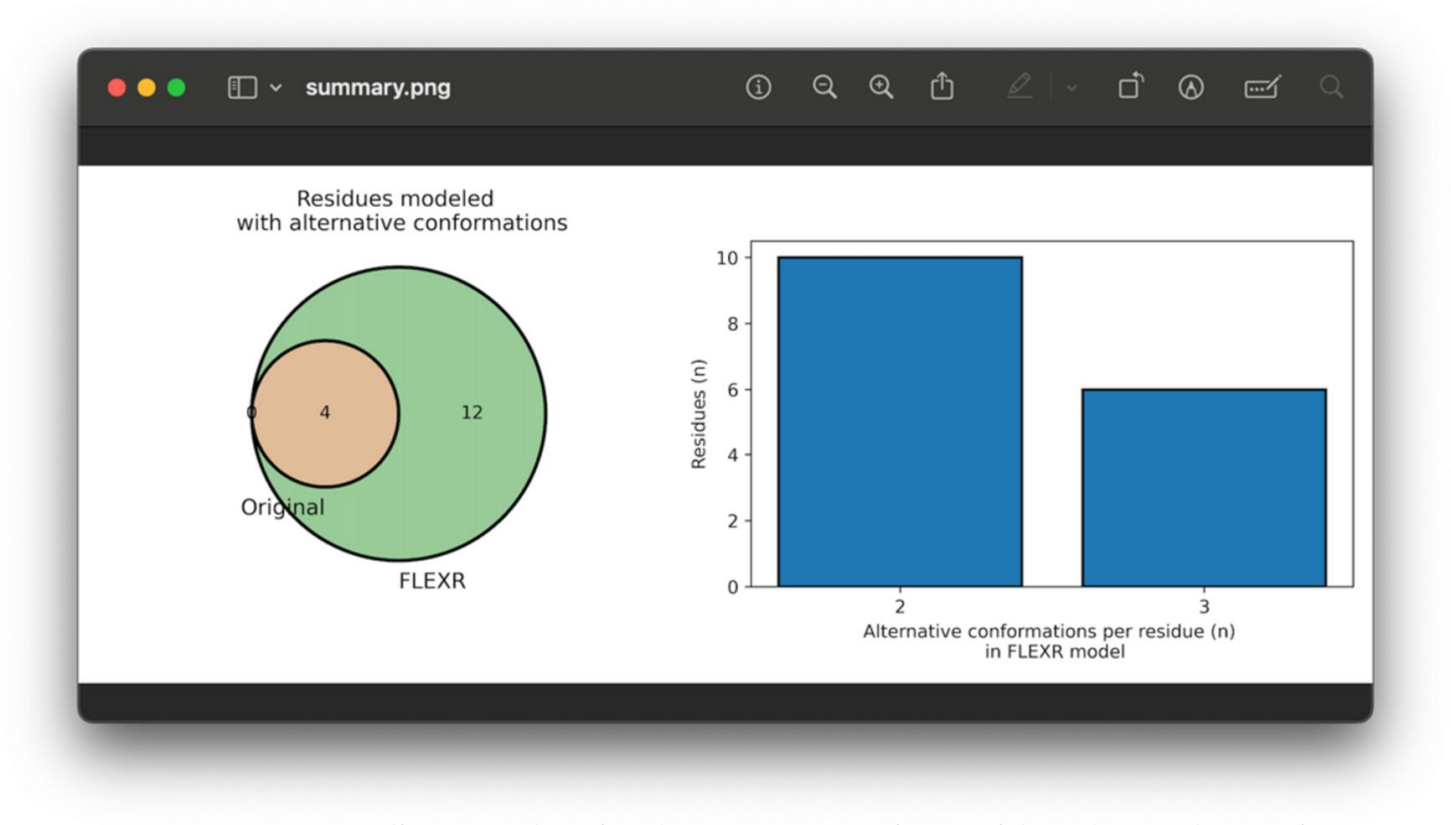
Summary plots produced by *FLEXR* for 8txg. A Venn diagram showing how often specific residues have alternative conformations modeled between the original model and the *FLEXR* model (left). A plot showing the number of alternative conformations per residue in the *FLEXR* model (right).

**Figure 4:**
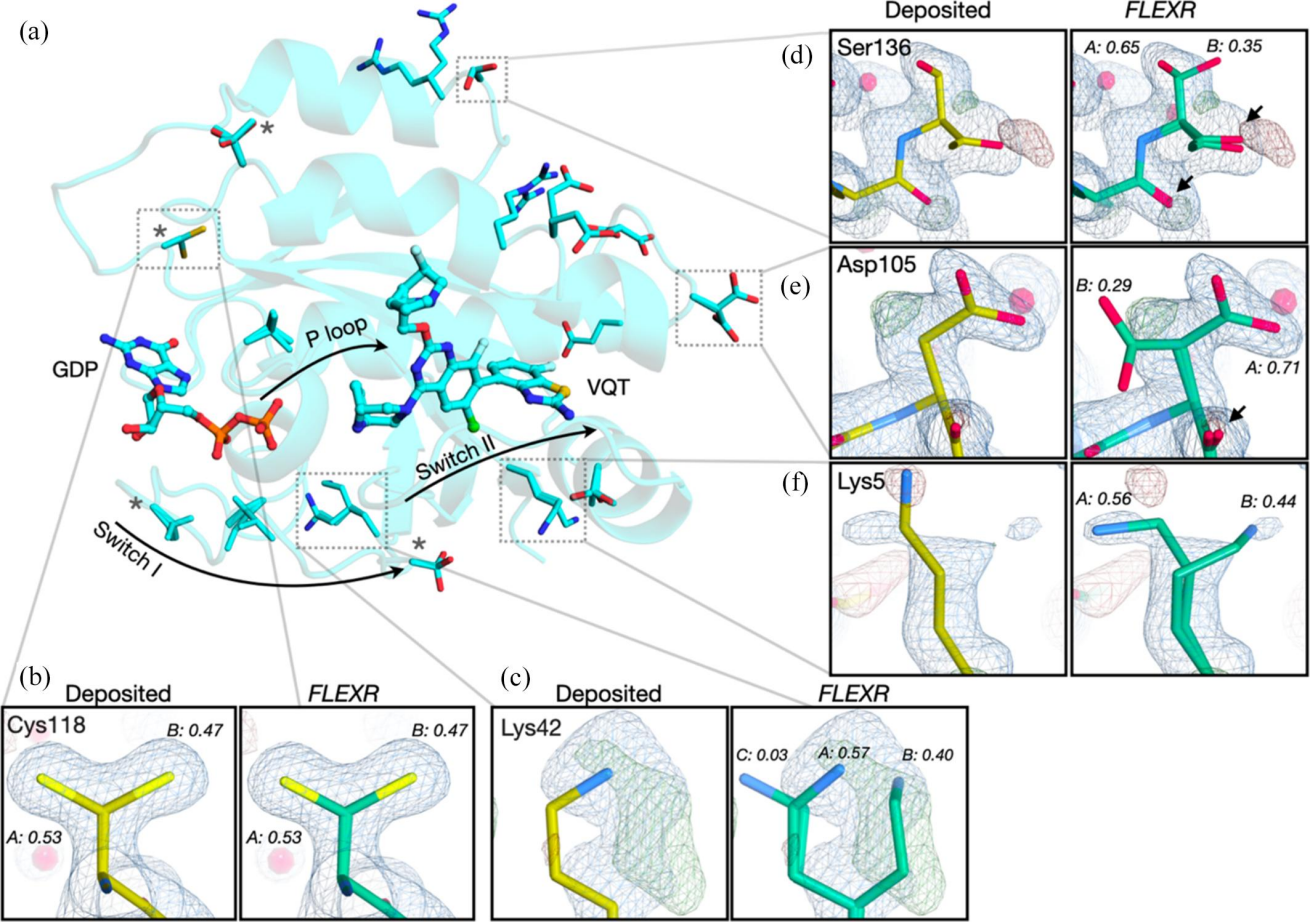
*FLEXR* reveals flexibility across KRAS-G12D. (*a*) Applying *FLEXR* to the KRAS-G12D structure (8txg, resolution 1.5 Å) revealed flexible sites along the Switch I and P-loop regions and adjacent to the Switch II region. This image was rendered in *PyMOL* (Schrödinger, New York). (*b*) Cys118 is an example of an alternative conformation that was present in the deposited model and reproduced by *FLEXR*. Examples of new conformational states include (*c*) Lys42, (*d*) Ser136, (*e*) Asp105 and (*f*) Lys5. Arrows in (*d*) and (*e*) indicate backbone conformational heterogeneity discovered by *FLEXR*. *F*_o_ − *F*_c_ maps are shown as green and red mesh and are contoured at ±3σ, respectively. 2*F*_o_ − *F*_c_ maps are shown as dark blue mesh and are contoured at 1σ. Maps shown here were calculated from the deposited model as provided by PDBe. Maps calculated from the refined *FLEXR* model are shown in Fig. S1.

The *FLEXR* model retained *R* values nearly identical to the re-refined deposited model (Table 1 ▸). The *FLEXR* model had no rotamer outliers but a heightened clashscore. Clashes like these might be from misplaced water molecules or rotamers (Table S1 and Fig. S2 of the supporting information) or water molecules that should be carefully modeled with alternative positions by the user (Richardson *et al.*, 2018 ▸). *FLEXR* reproduced flexibility at all 4 previously modeled multi-conformer sites and found an additional 12 sites that are missing in the deposition, totaling 16 total residues with alternative conformations [Figs. 3 ▸ and 4 ▸(*a*)]. Of these, 10 residues were modeled with 2 alternative conformations and 6 residues were modeled with 3 conformations (Fig. 3 ▸). One example of *FLEXR* reproducing a deposited alternative conformation is Cys118 [Fig. 4 ▸(*b*)]. *FLEXR* discovered new areas of flexibility throughout the structure, most notably at areas near the 2 binding sites [Fig. 4 ▸(*a*)], including Lys42 [Fig. 4 ▸(*c*)], Ser136 [Fig. 4 ▸(*d*)], Asp105 [Fig. 4 ▸(*e*)] and Lys5 [Fig. 4 ▸(*f*)]. *FLEXR* has the option to add atoms for alternative conformations beginning at the Cα atom or for all atoms in a residue, including backbone atoms. The utility of this is captured in the cases of Ser136 [Fig. 4 ▸(*d*)] and Asp105 [Fig. 4 ▸(*e*)]. In both cases, *FLEXR* found a second conformation and corresponding backbone conformational heterogeneity. A general benefit of the ability to build all atoms of the residue is that the strain of neighboring residues may be relieved upon refinement, leading to backbone heterogeneity and improved geometry (Stachowski & Fischer, 2023 ▸). For Lys5, the negative peak in the *F*_o_ − *F*_c_ map and insufficient 2*F*_o_ − *F*_c_ density suggest that the conformation in the deposited structure is incorrect [Fig. 4 ▸(*f*)]. *FLEXR* found an additional conformation at this position and the misplaced rotamer from the deposited model was corrected during refinement. Alternative conformations with low occupancies like Lys42 conformer C [Fig. 4 ▸(*c*)] should be removed from the model before deposition and the remaining two rotamers adjusted to better satisfy the density. Overall, *FLEXR* increased the structural heterogeneity of 8txg from ∼2 to ∼10% of residues being modeled as flexible.

## 4. Conclusions

Here we describe a GUI for *FLEXR* that is available as a *Coot 1* plugin. This approach has three advantages over the previously reported CLI version. First, the GUI connects users with a point-and-click approach to running *FLEXR*, which will accommodate those who are unfamiliar with command-line tools. Second, the GUI version integrates tools from *Phenix*, namely *Ringer*, to create a seamless workflow for producing multi-state models starting from a refined model. Third, implementing the GUI within the *Coot* framework means that models produced by *FLEXR* are immediately available for further inspection and analysis using the comprehensive suite of tools available in *Coot*. Using a recent KRAS structure, we illustrated how the *FLEXR* GUI can quickly and easily produce multi-state models. Future implementations will expand *FLEXR*’s compatibility with different map types and with performing single-conformer modeling. Ultimately, we anticipate that the *FLEXR* GUI will be a useful tool to retrospectively and prospectively interrogate protein structures for flexibility, which is often missed otherwise yet is key to understanding biology and informing ligand discovery and design.

## 5. Related literature

The following references are only cited in the supporting information for this article: Smart *et al.* (2012 ▸) and Yamashita *et al.* (2023 ▸).

## Supplementary Material

Supporting information. DOI: 10.1107/S1600576724001523/ei5111sup1.pdf

## Figures and Tables

**Table 1:** Quality metrics for deposited, re-refined and *FLEXR* models As an example, using the *FLEXR* GUI, we investigated a recently reported structure of human KRAS-G12D bound to an inhibitor (8txg, resolution 1.5 Å). After running *FLEXR* with default settings and re-refining with three macrocycles with default settings in *Phenix*, we obtained a final model with global quality metrics similar to those in the re-refined deposited model (Chen *et al.*, 2010 ▸).

	Deposited	Deposited-refined	*FLEXR*
*R*_work_	0.183	0.180	0.178
*R*_free_	0.205	0.210	0.208
*R*_gap_	0.022	0.030	0.030
Clashscore	1.42	1.78	6.07
Rotamer outliers (%)	0	0	0
Rama-*Z* (whole)	−0.37	−0.56	−0.53
