# Supplementary material for: FLEXR GUI: a graphical user interface for multi-conformer modeling of proteins
Source: J Appl Crystallogr. 2024 Mar 27;57(Pt 2):580–6. doi: 10.1107/S1600576724001523 (PMC11001397; doi:10.1107/S1600576724001523)
Supplement: Supplementary file 1 [file j-57-00580-sup1.pdf]

JOURNAL OF
APPLIED
CRYSTALLOGRAPHY

Volume 57 (2024)

Supporting information for article:

FLEXR GUI: a graphical user interface for multi-conformer modeling of proteins

Timothy R. Stachowski and Marcus Fischer

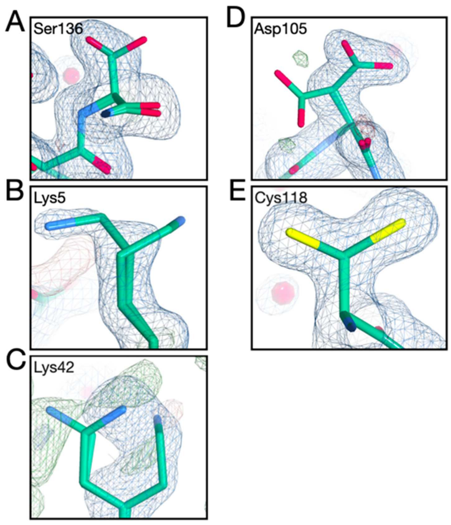

Figure S1 Maps calculated from the refined *FLEXR* model for examples of alternative conformations. Fo-Fc maps are shown as green and red mesh and contoured at +/- 3σ, respectively. 2Fo-Fc maps are shown as dark blue mesh and are contoured at 1σ.

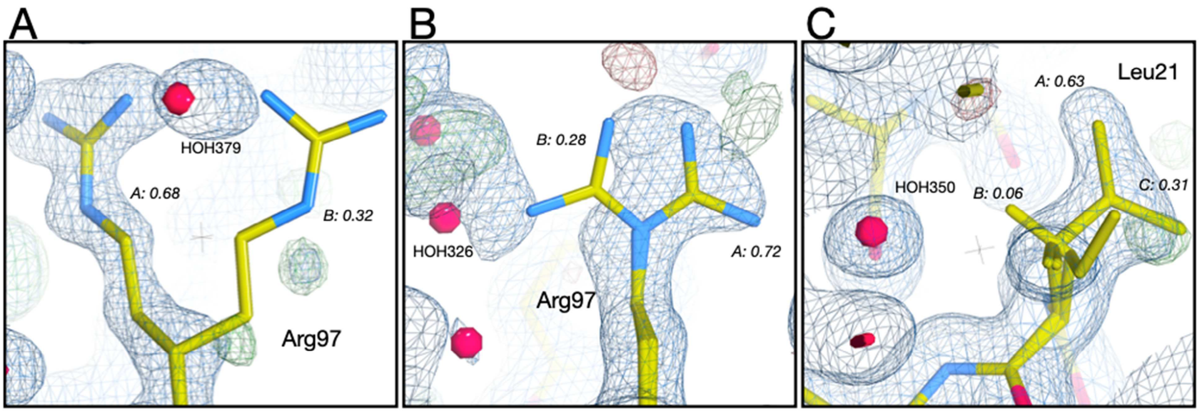

Figure S2 **Examples of alternative conformations clashing with water molecules from Table S1.** 2FoFc (contoured 1σ) and Fo-Fc (contoured at +/- 3σ) maps calculated from the deposited structure are shown for (a) Arg73, (b) Arg97, and (C) Leu21. Clashing waters are labeled.

Table S1 Top ten clashes in the refined *FLEXR* model calculated with 'phenix.clashscore'.

Clashing group 1					Clashing group 2					
**Chain**	**Residue number**	**Residue**	**altloc**	**Atom**	**Chain**	**Residue number**	**Residue**	**altloc**	**Atom**	**Overlap**
A	97	ARG	B	NH1	A	326	HOH		O	0.845
A	135	ARG	B	NH1	A	379	HOH		O	0.754
A	106	SER		OG	A	108	ASP	B	OD1	0.673
A	17	SER		O	A	21	ILE	B	HG22	0.661
A	98	GLU	B	OE1	A	99	GLN		NE2	0.637
A	21	ILE	B	HG21	A	29	VAL	B	HG22	0.634
A	21	ILE	B	HG23	A	350	HOH		O	0.587
A	142	ILE		HD12	A	155	ALA		HA	0.563
A	127	THR		HG22	A	131	GLN		HE21	0.535
A	71	TYR		O	A	74	THR	C	HG22	0.533

S1. Supplementary Methods

The *FLEXR* GUI was tested on Intel and silicon Macs running macOS Sonoma. You will need the following tools installed and accessible in your path:

1. Git (https://github.com/git-guides/install-git)
2. *Phenix* (Required to run Ringer) (https://phenix-online.org)
3. Homebrew (https://brew.sh)
4. *Coot* 1 (https://github.com/pemsley/coot)

S1.1. Installation

Once these are installed, you need to install these python libraries to the Homebrew Python:

1. Install Python libraries (approximate path for silicon Macs):

```
/opt/homebrew/bin/python3.12 -m pip install pandas numpy scipy
matplotlib matplotlib-venn --break-system-package
```

2. Clone the latest release of *FLEXR*-GUI:

```
git clone https://github.com/TheFischerLab/FLEXR-GUI.git
```

3. Move the contents of '/FLEXR-GUI' into the *Coot 1* Python directory. For example, on silicon Macs, where <version> is the *Coot 1* version you installed:

```
cp -r ./FLEXR-GUI/*
/opt/homebrew/Cellar/coot/<version>/lib/python3.12/site-
packages/coot
```

   and Intel Macs:

```
cp -r ./FLEXR-GUI/*
/usr/local/Cellar/coot/<version>/lib/python3.12/site-packages/coot/
```

4. Launch *Coot 1* with the *FLEXR*-GUI extension using:

```
/path/to/bin/coot --script /path/to/flexr_extentions.py
```

5. (optional) A variable in your path can be created so that *Coot 1* always opens with *FLEXR* loaded:

```
alias coot1='/path/to/bin/coot --script
/path/to/FLEXR_extentions.py'
```

S1.2. Usage

1. **Launching *FLEXR*.** When you launch *Coot 1* - you should see a button for the *FLEXR* menu:

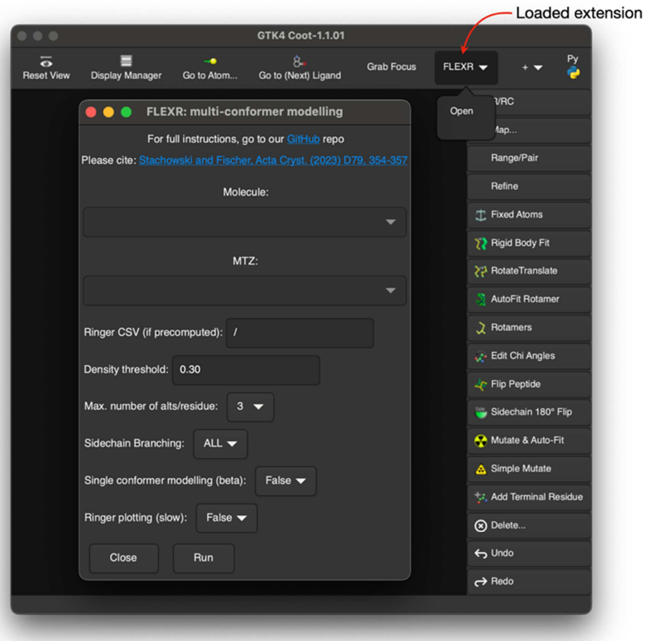

2. **Inputs.** *FLEXR* can be run in two ways - (a) with and (b) without pre-computed *Ringer* density values.

   a. If you want to run *FLEXR*, including *Ringer*, then you need: (1) a coordinate file (PDB or mmCIF), (2) map coefficients and reflections (MTZ), and (3) *Phenix* installed.

      i. The input MTZ file and model can be brought in from outside *Coot* (i.e. something you're working on locally on your computer) or fetched from the PDBe within *Coot*.

      ii. The MTZ and model will auto populate the 'Molecule' and 'MTZ' fields. If more than one map or model is present in the workspace, then specific ones can be chosen by clicking each input box, which will open a drop-down menu.

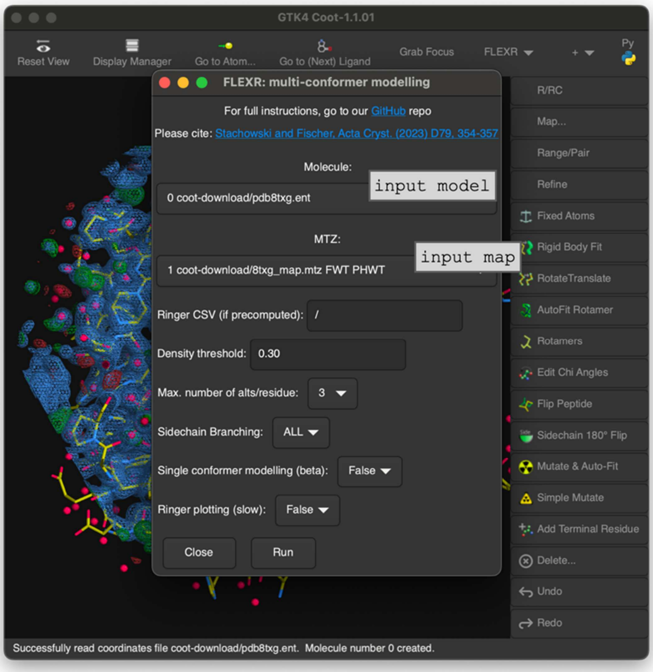

b. If you have pre-computed *Ringer* density values, you can use those by providing the full path to the *Ringer* CSV file. In this case, you do not need to supply an input MTZ file.

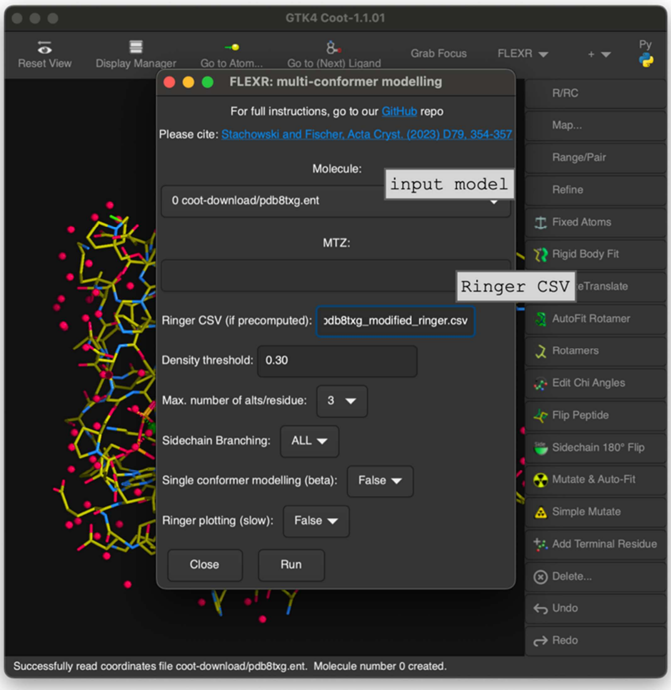

3. **Determining parameters.** These parameters apply to the entire structure.

   a. **Density threshold**. This is the map sigma (σ) value threshold used for detecting peaks in *Ringer* density values. Values above this level are treated as signal and values below are treated as noise. The default is 0.3σ, which was determined by Lang and colleagues (Lang *et al.*, 2010) to be the optimal value for structures with resolutions between 1.0-1.5 Å.

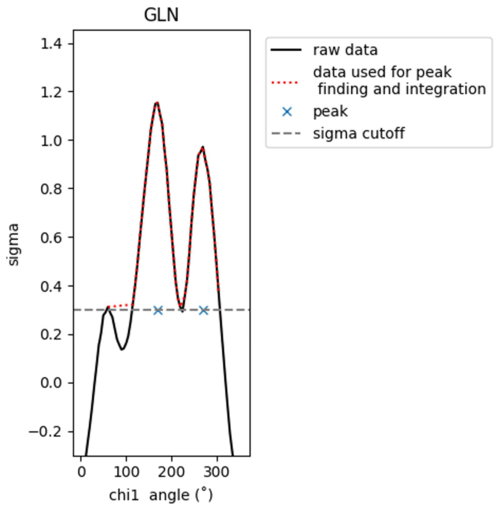

b. **Max. number of alts per residue**. This is the maximum number of alternative conformations built per residue. The default is 3; you can choose values between 2-10.

c. **Side chain branching**. This option determines whether atoms for an alternative conformation are added starting at the C-alpha atom or for the entire residue including the backbone. The default is to build the entire residue ('ALL') to enable alternative backbone conformations upon refining proximal alternative conformers.

d. **Ringer plotting**. This option produces Ringer plots (like the one above) for all dihedrals across all residues. These plots show the raw electron density values (black solid line) in addition to (1) values treated as signal (red dotted line) based on the threshold (step a.) and (2) areas of the density determined to be peaks (blue x's). The default is to not produce plots since it slows the program down and generates many files.

4. **Outputs.**

a. Within *Coot*, *FLEXR* will create two models:

i. One ending in '`_modified.pdb`': this is a single conformer model. This model takes the input model and removes any alternative conformations present. This is the template that *FLEXR* builds on. This model is generated with '`phenix.pdbtools`' using default settings.

ii. One ending in '`_FLEXR.pdb`': this is the *FLEXR* multi-conformer model.

b. Output files will also be produced in the directory from which *Coot* was launched:

i. '`log`' - log file with parameters used

ii. '`_ringer.csv`' - raw *Ringer* electron density values

iii. '`peak_finder....csv`' - *Ringer* peak detection summary

iv. '`_alts.csv`' - list of alternative conformations found by *FLEXR* that were built into the model

v. '`_FLEXR.pdb/cif`' - final output *FLEXR* model (in PDB and mmCIF formats) that should be used for further refinement.

vi. '`summary.png`' - some summary figures

vii. ‘`alts-original-flexr-comparison.txt`’ – list of residues with alternative conformations in *FLEXR* and original models.

5. **What’s next?**
   a. Since *FLEXR* only adds in rotamers with ideal geometry, users should do a quick refinement to better fit the model to the density. The quickest way to do this within *Coot* is performing real space refinement: Refine->RSR All Atoms.

   b. To inspect alternative conformations built by *FLEXR* there is a native ‘alt-conf’ GUI in *Coot 1*: Draw->Molecule->Residues with alt-confs… This is very handy to thumb through all alternative conformations and decide whether these are worth keeping, deleting, or refining.

   c. Occupancies can be estimated using your refinement program of choice such as *Phenix* (Liebschner et al., 2019), *REFMAC* (Yamashita et al., 2023)*, or BUSTER* (Smart et al., 2012). Alternative conformations with low occupancies (such as Lys conformer C in Fig. 4.C) should be removed from the model before deposition.

6. Command line interface. The CLI equivalent for the tutorial is:

```
phenix.pdbtools 8txg.pdb remove_alt_confs=True
phenix.maps 8txg_modified.pdb 8txg.mtz
mmtbx.ringer 8txg_modified.pdb 8txg_modified_map_coeffs.mtz
python flexr.py -f 8txg_modified_ringer.csv -pdb 8txg_modified.pdb -
build True
```
